# Persisting symptoms after *Cryptosporidium hominis* outbreak: a 10-year follow-up from Östersund, Sweden

**DOI:** 10.1007/s00436-023-07866-8

**Published:** 2023-05-18

**Authors:** Marije Boks, Mikael Lilja, Micael Widerström, Pontus Karling, Anna Lindam, Malin Sjöström

**Affiliations:** 1 Department of Public Health and Clinical Medicine, Umeå University, Umeå, Sweden; 2 Unit of Research, Education and Development – Östersund, Department of Public Health and Clinical Medicine, Umeå University, Umeå, Sweden; 3 Department of Clinical Microbiology, Umeå University, Umeå, Sweden

**Keywords:** *Cryptosporidium*, Disease outbreaks, Sequelae, Post-infectious symptoms, PI-IBS, Diarrhoea

## Abstract

In late 2010, an outbreak of *Cryptosporidium hominis* affected 27,000 inhabitants (45%) of Östersund, Sweden. Previous research shows that abdomen and joint symptoms commonly persist up to 5 years post-infection. It is unknown whether *Cryptosporidium* is associated with sequelae for a longer duration, how persisting symptoms present over time, and whether sequelae are associated with prolonged infection. In this prospective cohort study, a randomly selected cohort in Östersund was surveyed about cryptosporidiosis symptoms in 2011 (response rate 69.2%). A case was defined as a respondent reporting new diarrhoea episodes during the outbreak. Follow-up questionnaires were sent after 5 and 10 years. Logistic regressions were used to examine associations between case status and symptoms reported after 10 years, with results presented as adjusted odds ratios (aOR) with 95% confidence intervals. Consistency of symptoms and associations with case status and number of days with symptoms during outbreak were analysed using *X*^2^ and Mann–Whitney *U* tests. The response rate after 10 years was 74% (*n* = 538). Case status was associated with reporting symptoms, with aOR of ~3 for abdominal symptoms and ~2 for joint symptoms. Cases were more likely to report consistent symptoms. Cases with consistent abdominal symptoms at follow-up reported 9.2 days with symptoms during the outbreak (SD 8.1), compared to 6.6 days (SD 6.1) for cases reporting varying or no symptoms (*p* = 0.003). We conclude that cryptosporidiosis was associated with an up to threefold risk for reporting symptoms 10 years post-infection. Consistent symptoms were associated with prolonged infection.

**Supplementary Information:**

The online version contains supplementary material available at 10.1007/s00436-023-07866-8.

## Background

*Cryptosporidium* is a protozoan that causes diarrhoea among humans and animals (Pogreba-Brown et al. 2020). Over 90% of human cases are caused by the species *Cryptosporidium hominis* and *C. parvum*, usually following intake of contaminated water or food or sometimes through direct contact with an infected individual or animal (Bouzid et al. 2013; Chalmers and Davies 2010; Feng et al. 2018). Infection usually causes diarrhoea, abdominal pain, vomiting, and/or fever but can sometimes be asymptomatic. Cryptosporidiosis is self-limiting in immunocompetent individuals but can sometimes be prolonged (≥7 days) or persistent (≥15 days), with diarrhoea lasting up to 3 weeks (Chalmers and Davies 2010; Checkley et al. 2015). Recurrence of gastrointestinal symptoms after several asymptomatic days is common, even in immunocompetent individuals (Hunter et al. 2004; MacKenzie et al. 1995).

Despite being a common cause of diarrhoea worldwide, only a few large *Cryptosporidium* outbreaks have been reported in the literature (Efstratiou et al. 2017; MacKenzie et al. 1995; Widerström et al. 2014). In November 2010, the city of Östersund, Sweden, was affected by an outbreak of *C. hominis* IbA10G2 due to contamination of the public water supply. An estimated 27,000 individuals, constituting approximately 45% of the city’s population, reported symptoms of cryptosporidiosis (Widerström et al. 2014).

After gastroenteritis, sequelae are common and can include post-infectious irritable bowel syndrome (PI-IBS), reactive arthritis, and joint pain (Pogreba-Brown et al. 2020). PI-IBS development is considered to be multifactorial: altered signalling by the gut–brain axis, dysbiosis in the intestinal flora, abnormal visceral pain signalling, and intestinal immune activation are contributing factors (Aguilera-Lizarraga et al. 2022). Younger individuals, women, and persons with severe enteritis are more likely to develop PI-IBS (Berumen et al. 2021a). Regarding IBS in general, the presence of anxiety and depression are known to double the risk of experiencing and maintaining symptoms (Sibelli et al. 2016).

Several microorganisms, including the most common gastroenteritis-causing bacteria, and parasites such as *Amoeba* spp. and *Toxoplasma gondii*, are also associated with an increased risk of inflammatory bowel disease (IBD) (Axelrad et al. 2020). After the *Cryptosporidium* outbreak in 2010, an increase of late-onset IBD and microscopic colitis was noted in Östersund (Boks et al. 2022).

Persisting sequelae after *Cryptosporidium* infections are common and may include diarrhoea, abdominal pain, fatigue, and headache (Carter et al. 2020; Carter et al. 2019; Hunter et al. 2004; Iglói et al. 2018). We previously reported that individuals from Östersund, who reported cryptosporidiosis symptoms, were more commonly affected by abdominal symptoms, fatigue, headache, and joint-related symptoms up to 5 years after the outbreak (Lilja et al. 2018; Sjöström et al. 2022). There is a lack of studies regarding the existence of sequelae more than 5 years after the initial infection. Therefore, in the present study, we aimed to evaluate whether post-infectious symptoms persisted at 10 years after *Cryptosporidium* infection and, if so, how they presented over time. Our secondary aims were to investigate whether long-term persisting symptoms were associated with the duration of the primary infection and whether persistent symptoms were related to self-reported health concerns at the time of the outbreak.

## Methods

We performed a prospective cohort study, with follow-up of adult inhabitants of Östersund after the *C. hominis* outbreak in November 2010.

### Study population and data collection

In January 2011, 2 months after the start of the outbreak, 1524 randomly selected inhabitants of Östersund municipality, including 1215 adults (born 1992 or earlier), were invited to complete a written questionnaire (outbreak questionnaire) sent to them by post. The questionnaire included items regarding demographics, cryptosporidiosis symptoms, health concerns in relation to the outbreak, and medical conditions, including pre-existing gastrointestinal disease. The questionnaire was returned by 1044 inhabitants (69.2%), of whom 53.4% were women. The response rate was the lowest among young adults (20–29 years old, 48.8%) and the highest among individuals >60 years of age (87.2–89.2%) (Widerström et al. 2014). Follow-up surveys were administered after 6 months, 2 years, and 5 years, by sending a questionnaire regarding possible sequelae to all individuals who responded to the outbreak questionnaire (Lilja et al. 2018; Rehn et al. 2015; Sjöström et al. 2022).

**Fig. 1:**
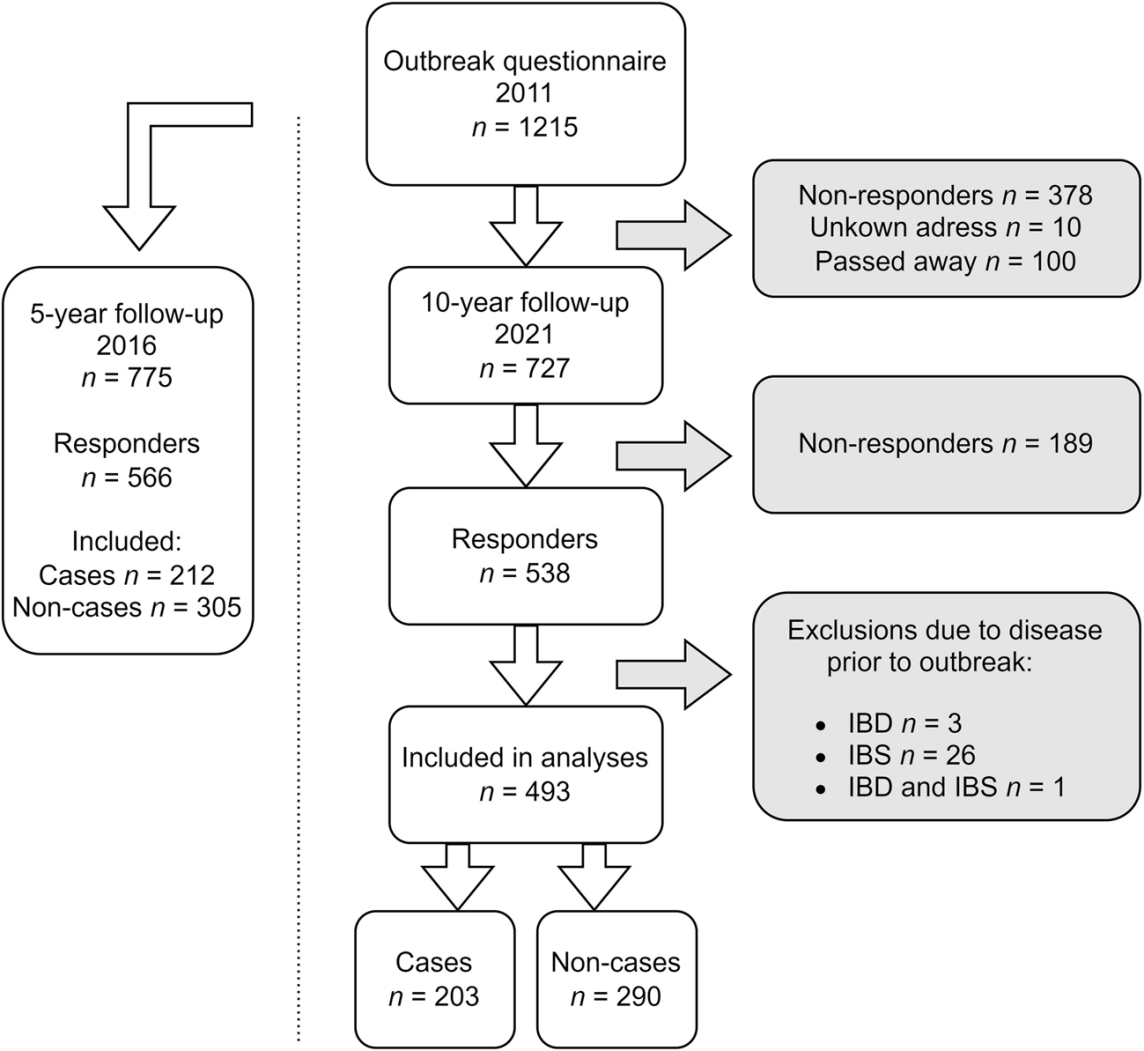
Case selection. A case was defined as a respondent who reported new episodes of diarrhoea (≥3 loose stools per day) and/or watery diarrhoea between November 1, 2010, and January 21, 2011, and who lived in Östersund municipality mid-January 2011. A non-case was defined as a respondent not fulfilling the case criteria. IBD, inflammatory bowel disease. IBS, irritable bowel syndrome

For the present study, in January 2021, a new follow-up questionnaire (10-year follow-up questionnaire, Supplement 1) was sent by post to the 727 adults who had replied to the outbreak questionnaire in 2011 (Fig. 1). The mailing included a pre-paid envelope to return the questionnaire, and a reminder was sent after 1 month. The respondents were asked to report whether they had experienced the following possible post-infectious symptoms during the last 3 months: loose stools, watery diarrhoea, bloody diarrhoea, change in bowel habits, abdominal pain, bloating, nausea, vomiting, heartburn, loss of appetite, weight loss, headache, eye pain, fatigue, stiff joints, joint pain, swollen joints, and/or joint discomfort. They were also asked to report any other symptoms, and the extent to which they had concerns regarding their health. The questionnaire also included questions regarding the presence of the following conditions: IBS, IBD, gluten intolerance, lactose intolerance, other persisting abdominal issues, ulcers, diabetes, chronic lung diseases, heart failure, rheumatic disease, and cancer. Finally, we asked about the participants’ use of antacids, systemic corticosteroids, and immunomodulating drugs. The returned questionnaires were optically scanned and then transformed to a data file.

#### Exclusion

Respondents were excluded if they had a (self-reported) diagnosis of IBD, IBS, or other long-term bowel issues prior to the outbreak.

#### Case definition

A case was defined as a respondent who, on the outbreak questionnaire, reported new episodes of diarrhoea (≥3 loose stools daily) and/or watery diarrhoea with onset between November 1, 2010, and January 31, 2011 (World Health Organisation 2017). Respondents who did not fulfil these criteria were defined as non-cases.

### Outcome measures

All reported symptoms, and their association with case status, were analysed separately. We also created composite outcomes, where there was considerable overlap in symptoms reported: *abdominal symptoms* included loose stools, watery and bloody diarrhoea, abdominal pain, constipation, changing bowel habits, and bloating; *diarrhoea* included loose stools, watery diarrhoea, and bloody diarrhoea; and *joint symptoms* included joint discomfort, stiff joints, joint pain, and swollen joints.

*Medical conditions* and use of *medication* were solely based on the participants’ answers on the questionnaires.

*Symptoms over time* were explored by merging the data from the 5- and 10-year follow-ups. We used the composite outcomes abdominal symptoms and joint symptoms, as well as fatigue and headache and divided the participants into three categories: *consistent* symptoms (the symptom was reported in both the 5- and 10-year follow-up); *varying* symptoms (the symptom was reported once, either in the 5- or 10-year follow-up); and *no symptoms* in either of the follow-ups.

*Prolonged disease* was determined based on the number of days with abdominal symptoms, as reported on the outbreak questionnaire. Prolonged disease was defined as ≥8 days of abdominal symptoms at the time of the outbreak.

In the outbreak questionnaire, participants rated their *concerns* about their own health in relation to the outbreak on a single scale ranging from 0 to 10, with 0 indicating no concerns at all, and 10 being very worried. The scale was converted to a dichotomous variable with ≥7/10 as the cut-off point for significant symptoms. This decision was based on the Hospital Anxiety and Depression Scale (HADS) (Zigmond and Snaith 1983) and similar scales on which significant symptoms are defined based on a score of at least 50–75% of the maximum score.

### Statistical analyses

The study population was stratified according to case status, sex, and age at the time of the outbreak (18–40, 41–65, and ≥66 years). *X*^2^ tests and Mann–Whitney *U* tests were used to assess between-group differences in demographic variables and mean number of symptoms. We applied logistic regressions to examine associations between case status and symptoms reported on the follow-up questionnaire. The results were adjusted for age (category) and sex and presented as adjusted odds ratio (aOR) with 95% confidence interval (CI). *X*^2^ tests were used to evaluate possible association between prolonged disease during the outbreak and reported symptoms after 10 years, as well as to analyse the consistency of symptoms and their association with case status. The Mann–Whitney *U* test was used to examine an association between consistent symptoms and the mean number of days with symptoms during the outbreak. The dichotomous variable for health concerns was used to evaluate a possible association with the symptoms reported during follow-up, using logistic regression and with adjustment for age and sex. All statistical calculations were performed using IBM SPSS Statistics for Windows, version 28 (IBM Corp., Armonk, NY, USA). Missing values were excluded from analysis. The significance level was set at 0.05.

## Results

A total of 538 adults (74.0%) responded to the 10-year follow-up questionnaire. Response rates were the lowest (60.1%) among those who were 18–40 years old during the outbreak and the highest (81.1%) among those aged 41–65 years. Case status and sex did not differ between responders and non-responders (data not shown).

After exclusion of individuals who had IBD, IBS, or other long-term bowel issues prior to the outbreak, 493 individuals were included in the final analyses (Fig. 1), including 203 respondents defined as cases and 290 as non-cases. There were no sex differences between the groups. The median age at the time of the outbreak was 47.8 years (range 18–80 years) for cases and 52.7 years (18–89 years) for non-cases (*p* ≤ 0.001). Table 1 presents the detailed demographic information. Cases and non-cases did not differ in current smoking status or self-reported use of antacids, systemic corticosteroids, or immunomodulating agents. History of gastric ulcers was reported by no patients in the case group but 10 patients (3.6%) in the non-case group (*p* = 0.007). No other differences in comorbidities were found.

**Table 1:** Demographic characteristics of the study population

	Cases, *n* (%)	Non-cases, *n* (%)	*p* value
Total cases	203 (41.2)	290 (58.8)	
Sex			0.940
Male	91 (44.8)	131 (45.2)	
Female	112 (55.2)	159 (54.8)	
Age at outbreak, years			0.034
18–40	67 (33.0)	70 (24.1)	
41–65	106 (52.2)	156 (53.8)	
≥66	30 (14.8)	64 (22.1)	

### Reported symptoms after 10 years

Ten years after the outbreak, at least one symptom was reported by 59.6% of cases and 45.1% of non-cases. The mean number of symptoms among cases was 3.0 (range 0–15, median 2), while non-cases reported a mean of 1.7 symptoms (range 0–16, median 0) (*p* ≤ 0.001). The more frequently reported symptoms in the case group were bloating, headache, fatigue, and joint discomfort. Compared to the non-case group and corrected for age and sex, cases were significantly more likely to report abdominal symptoms (e.g. diarrhoea, changes in bowel habits, abdominal pain, nausea, bloating and heartburn); joint symptoms (e.g. joint pain, joint stiffness, and joint discomfort); and headaches, fatigue, and loss of appetite (Table 2). These results were not altered when the analyses were corrected for self-reported lactose intolerance and gluten intolerance (for abdominal symptoms) or rheumatic disease (for joint symptoms) at baseline (data not shown).

**Table 2:** Symptoms reported 10 years after the outbreak, presented for cases and non-cases

Outcomes		Cases, *n* (%)	Non-cases, *n* (%)	aOR (95% CI)
Diarrhoea^a^		36 (18.1)	20 (6.9)	2.9 (1.6–5.2)*
	Loose stools	27 (13.3)	14 (4.8)	3.1 (1.6–6.0)*
	Watery diarrhoea	21 (10.3)	13 (4.5)	2.4 (1.1–4.9)*
	Bloody diarrhoea	8 (3.9)	2 (0.7)	-
Constipation		28 (13.8)	28 (9.7)	1.5 (0.9–2.7)
Changes in bowel habits		42 (20.7)	24 (8.3)	2.9 (1.7–5.0)*
Abdominal pain		38 (18.7)	20 (6.9)	3.0 (1.7–5.4)*
Bloating		79 (38.9)	54 (18.6)	2.8 (1.8–4.2)*
Heartburn		41 (20.2)	34 (11.7)	2.0 (1.2–3.2)*
Nausea		29 (14.3)	20 (6.9)	2.1 (1.1–3.9)*
Vomiting		7 (3.4)	5 (1.7)	1.8 (0.6–5.9)
Joint symptoms^a^		73 (36.5)	68 (23.6)	1.9 (1.3–2.9)*
	Joint discomfort	59 (29.1)	45 (15.5)	2.3 (1.5–3.6)*
	Stiff joints	54 (26.6)	43 (14.8)	2.2 (1.4–3.4)*
	Joint pain	54 (26.6)	54 (18.6)	1.6 (1.1–2.5)*
	Swollen joints	23 (11.3)	26 (9.0)	1.4 (0.8–2.6)
Headache		64 (31.5)	57 (19.7)	1.7 (1.1–2.7)*
Ocular pain		28 (13.8)	22 (7.6)	1.9 (1.0–3.5)*
Fatigue		58 (28.6)	50 (17.2)	1.8 (1.2–2.8)*
Fever		12 (5.9)	8 (2.8)	2.1 (0.9–5.4)
Loss of appetite		13 (6.4)	5 (1.7)	4.2 (1.5–12.0)*
Weight loss		7 (3.4)	9 (3.1)	1.5 (0.5–4.2)

^a^Composite outcome: individuals reporting at least one of the symptoms in this category

Logistic regression analyses corrected for sex and age. *aOR* adjusted odds ratio. *CI* confidence interval

^*^Statistically significant

Among the cases, those who reported abdominal symptoms for ≥8 days on the outbreak questionnaire were more likely to report abdominal symptoms after 10 years (52.2%), compared to those who reported initial symptoms lasting up to 1 week (39.7%, *p* = 0.022). In 2021, more cases than non-cases cases reported a diagnosis of IBS (9.3% vs. 2.9%, *p* = 0.003) and lactose intolerance (11.2% vs. 6.1%, *p* = 0.048). Cases and non-cases did not differ in their reported use of medication.

### Symptoms over time

**Fig. 2:**
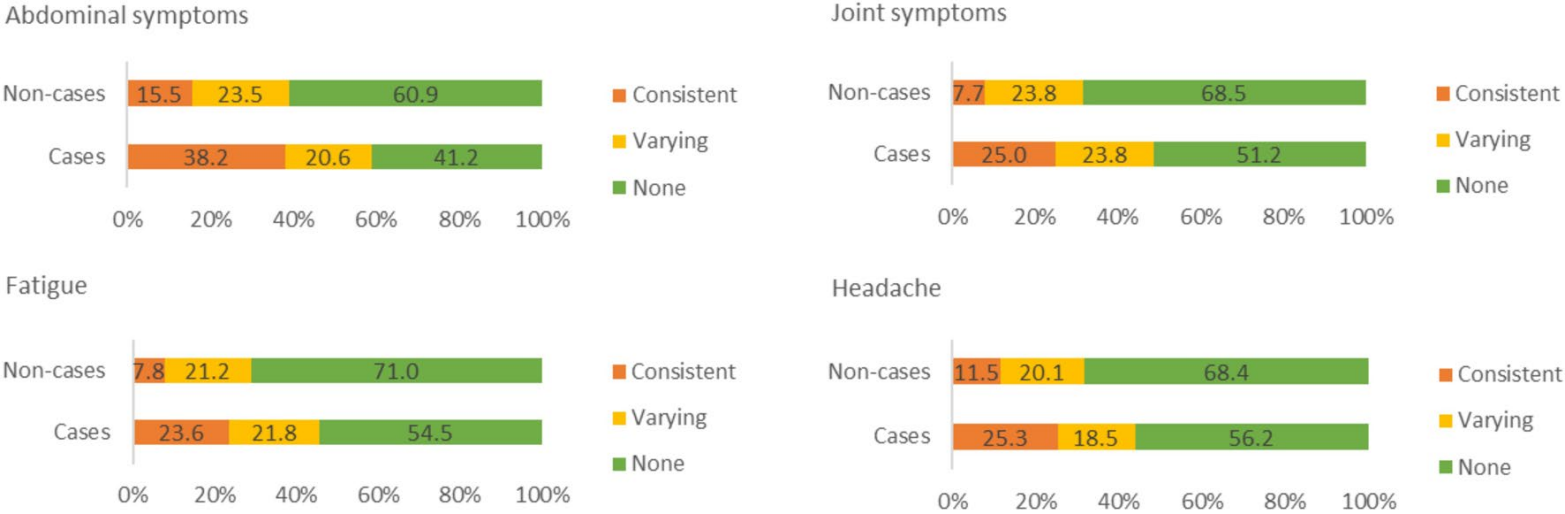
Consistency of symptoms over time, as reported by cases and non-cases. Percentages of participants reporting consistent, varying, or no symptoms, among cases and non-cases. Consistent: symptom reported after 5 *and* 10 years. Varying: symptom reported only after 5 *or* 10 years. None: symptom not reported. Abdominal symptoms: loose stools, watery or bloody diarrhoea, abdominal pain, constipation, changing bowel habits, and/or bloating. Joint symptoms: joint discomfort, joint pain, stiff joints, and/or swollen joints. *X*^2^ tests. Abdominal symptoms, joint symptoms, fatigue: *p* ≤ 0.001. Headache: *p* = 0.002

Among persons who responded to the 10-year follow-up questionnaire, 173 cases and 241 non-cases also replied to the 5-year follow-up questionnaire. Consistent symptoms were more commonly reported by cases compared to non-cases (Fig. 2). Among cases who reported consistent abdominal symptoms during both follow-ups, the mean duration of symptoms during the outbreak was 9.2 days (SD 8.1), compared to 6.6 days (SD 6.1) among cases reporting varying or no symptoms at follow-up (*p* = 0.003). Among those reporting varying symptoms, more participants tended to report symptoms during the 5-year follow-up compared to the 10-year follow-up, although this difference was not significant.

### Health concerns

As expected, cases had more concerns regarding their health at the time of the outbreak, with 13.3% reporting significant health concerns, compared to 2.5% of non-cases (*p* ≤ 0.001). Among cases, those with significant concerns about their health reported more days with abdominal symptoms during the outbreak (mean 12.3 days, SD 7.9), compared to those without significant concerns (mean 7.0 days, SD 6.6) (*p* ≤ 0.001). Cases that had significant health concerns at the time of the outbreak tended to more commonly report symptoms after 10 years (63.3% vs. 50.5%). Correcting for health concerns at baseline in the logistic regression analyses only attenuated the results slightly. Compared to cases without significant health concerns during the outbreak, cases with significant health concerns did not report more significant concerns about their health during the 10-year follow-up.

## Discussion

In this prospective cohort study, we evaluated the persistence of post-infectious symptoms 10 years after a large outbreak of *C. hominis* in Östersund, Sweden. Compared to non-cases, cases had a threefold greater risk of abdominal symptoms, and a twofold greater risk of joint symptoms, and more commonly reported having IBS and lactose intolerance diagnosed after the outbreak. Additionally, cases were more likely to report consistent symptoms over time. Among cases, those with prolonged disease during the outbreak were more likely to report symptoms 10 years later. Health concerns at baseline were not an independent risk factor for reporting sequelae during follow-up.

### Results in context

To our knowledge, this is the first report of persisting post-infectious symptoms a decade after *Cryptosporidium* infection. The present results are in line with previous follow-ups of the same population (Lilja et al. 2018; Rehn et al. 2015; Sjöström et al. 2022). Long-term abdominal symptoms have also been reported in another Swedish study that included 271 individuals with laboratory-confirmed cryptosporidiosis. After up to 36 months, 15% experienced episodes of diarrhoea and 9% abdominal pain (Insulander et al. 2013). In a systematic review based on pooled estimates from eight studies, with a follow-up time of 2–36 months, the most common sequelae after cryptosporidiosis were diarrhoea (25%), abdominal pain (25%), nausea (24%), fatigue (25%), and headache (21%). In accordance with our present findings, that review showed that abdominal pain, loss of appetite, fatigue, vomiting, joint pain, headache, and eye pain were 2–3 times more likely to occur after a *Cryptosporidium* infection (Carter et al. 2020). A large Norwegian study following an outbreak with another parasite, *Giardia lamblia*, revealed that infection was associated with abdominal symptoms and chronic fatigue 10 years later (Litleskare et al. 2018). Even though the numbers of individuals reporting post-infectious symptoms are striking, it should be noted that we do not know to what extent these symptoms affect patients in their daily lives.

Acute gastroenteritis is associated with increased risk of IBS. A large meta-analysis showed that this relationship applies to infections with bacteria (e.g. *Campylobacter spp.*, *Salmonella spp.*, and *Escherichia coli*) but also for norovirus and the protozoan *Giardia lamblia* (Klem et al. 2017). The authors found a general PI-IBS prevalence of 11%, with the highest risk after protozoal infection (40%). Notably, these numbers were based on only one cohort, comprising patients with laboratory-confirmed *G. lamblia* infection (Hanevik et al. 2009; Hanevik et al. 2014; Wensaas et al. 2012). From the same cohort, a 10-year follow-up was published, reporting an IBS prevalence of 43% among cases and 14% among controls (OR 4.7, CI 3.6–6.2) and chronic fatigue in 26% vs. 11% (OR 3.0, CI 2.2–4.1) (Litleskare et al. 2018). Their findings strengthen the hypothesis that protozoan infection can result in long-term abdominal complaints.

A recent study demonstrated a 21% prevalence of PI-IBS at 6–9 months after *Campylobacter* infection, with an additional 9% suffering from new-onset abdominal pain and/or bowel disturbances that did not meet the Rome criteria (Berumen et al. 2021b). This suggests that the actual burden of chronic abdominal sequelae after gastrointestinal infections might be greater than what is captured by a PI-IBS diagnosis based on the Rome criteria.

It is likely that many of the individuals reporting abdominal symptoms in our study suffer from PI-IBS, but we cannot fully diagnose them based on our data. A validated questionnaire based on the Rome IV criteria is available for diagnosis, but it is extensive (Palsson et al. 2016). To improve the response rate, we did not include this questionnaire in our study, but the questions regarding abdominal symptoms were based on the criteria. Therefore, we can only conclude that some of our participants suffered from post-infectious IBS-like symptoms. Our results are strengthened by the finding that cases reported IBS diagnosis after the outbreak more often than non-cases (9.3% vs. 2.9%, *p* = 0.003). Cases also more commonly reported lactose intolerance, which is in line with a meta-analysis showing an OR of 3.5 (CI 1.6–7.6) for IBS patients compared to healthy controls (Varjú et al. 2019).

PI-IBS development is more common among younger individuals, women, and persons with severe disease (Berumen et al. 2021a; Klem et al. 2017). In a large meta-analysis based on 8 studies, diarrhoea lasting >7 days was associated with increased odds of PI-IBS (OR 2.62, 95% CI 1.48–4.61). Accordingly, in our present study, we found that individuals who reported at least 8 days of abdominal symptoms during their acute infection more commonly experienced abdominal symptoms during follow-up, compared to persons whose acute symptoms lasted up to one week (52.2% vs. 39.7%). Additionally, compared to cases who did not seem to have chronic symptoms, the cases who reported abdominal symptoms after both 5 and 10 years had experienced more symptomatic days during their acute infection (6.62 vs. 9.19 days).

Among those who reported varying symptoms during the follow-ups, more participants tended to report symptoms after 5 years than after 10 years; however, this difference was not significant. This trend might suggest that some individuals can recover from their ailments even after several years.

Anxiety and depression double the risk of IBS development and contribute to the maintenance of symptoms (Sibelli et al. 2016). To improve the response rate, our study did not include any validated questionnaire. Our results did not show that health concerns were an independent predictive factor for sequelae. We assume that the public largely considered cryptosporidiosis to be an annoying but harmless infection not to be worried about. Notably, one question about health concerns cannot replace an anxiety or depression diagnosis; therefore, it is not possible to draw further conclusions based on these results.

### Strengths and limitations

A strength of this study was its prospective design, following a cohort that was randomly selected shortly after the outbreak. The research team had broad knowledge in general practice, infectious diseases, and gastroenterology and was supported by an experienced statistician. We obtained high response rates for the outbreak questionnaire and for the follow-up questionnaires, without relevant differences between cases and non-cases. Östersund is the only city in a fairly isolated region; therefore, we expect that few other factors would have influenced the outcomes on a population level.

A limitation of this study was that cases were defined based on self-reported symptoms. During the outbreak, only 149 inhabitants had their *Cryptosporidium* infection confirmed by faecal microscopy. The strain identified in the stool samples was the same as in the drinking water, and no other pathogens were identified during microscopy (Widerström et al. 2014). Therefore, it is reasonable to assume the symptoms reported by our population were caused by *C. hominis* IbA10G2. However, relying on self-reported symptoms might have enabled misclassification of status for some individuals. Notably, we expect that this could be the case for individuals in both groups, and thus, we do not expect it to have affected the results. Moreover, to minimise the risk of misclassifying individuals with chronic diarrhoea as cases, and thereby overestimating associations between diarrhoea and case status, we excluded all individuals with long-term abdominal issues, including IBS and IBD, before the outbreak.

We did not have information about the participants’ current infection status at the time of follow-up. However, it is unlikely that respondents had a chronic *Cryptosporidium* infection causing long-term symptoms. At the time of the 2-year follow-up, participants were invited to submit stool samples, and all returned samples were negative for *Cryptosporidium* spp. (Lilja et al. 2018). Since the 10-year follow-up was conducted after the start of the COVID-19 pandemic, it is possible that some reported symptoms were the result of a SARS-CoV-2 infection rather than long-term symptoms after a *Cryptosporidium* infection. However, this should have been equally possible for individuals in both groups and thus cannot explain the differences found.

With an increasing number of analyses, the risk of mass significance also increases. However, our results were consistently similar, which rather strengthens the outcomes.

### Future research and clinical implications

Early recognition and appropriate management of post-infectious IBS may improve patient outcomes and reduce healthcare costs. Further research is needed to better understand the long-term effects of *Cryptosporidium* infection on gastrointestinal and joint symptoms, including the potential development of IBS. Additionally, the long-term effects of *Cryptosporidium* infection on children should be explored. It would also be beneficial to investigate the mechanisms underlying the persistence of symptoms and to develop effective treatment strategies for those affected.

## Conclusions

Clinical cryptosporidiosis in adults was associated with a threefold risk of abdominal symptoms and a twofold risk of joint symptoms at 10 years after the initial infection. It is likely that many of the individuals reporting long-term abdominal symptoms had developed PI-IBS. An initial symptom duration of over 7 days during the acute infection was associated with an increased prevalence of long-term symptoms. Health concerns during the outbreak were not an independent risk factor. These results suggest that water treatment to prevent *Cryptosporidium* infections should be a priority for policy makers.

## Supplementary information

ESM 1

## Data Availability

The datasets used for the current study are available from the corresponding author upon reasonable request.
